# Effect and safety of traditional Chinese exercises for patients with type 2 diabetes: A protocol for systematic review and network meta-analysis

**DOI:** 10.1097/MD.0000000000028365

**Published:** 2021-12-23

**Authors:** Lijuan Zou, Linfeng Lei, Chuifeng Kong, Peiying Yu, Jiazhou Li, Hua-shan Pan

**Affiliations:** a School of physical education and health of Guangzhou University of Chinese Medicine, China; b Guangdong Chaozhou Health Vocational College, China.

**Keywords:** network meta-analysis, protocol, systematic review, traditional Chinese exercises, type 2 diabetes

## Abstract

**Background:**

Traditional Chinese exercises are more and more popular for type 2 diabetes patients for the treatment and rehabilitation; however, the comparative effectiveness and safety remains unclear. Our study aims to compare the pros and cons of these exercise interventions for type 2 diabetes by implementing a network meta-analysis.

**Methods:**

Eight databases will be searched for relevant systematic reviews including SinoMed, VIP, CNKI, Wanfang, PubMed, Embase, Web of Science and the Cochrane Library from inception to Oct 2021. Randomized controlled trials that meeting eligibility in published systematic reviews will be identified. Randomized controlled trial related to Traditional Chinese Exercises or Qigong therapy in the treatment of type 2 diabetes will be included. Two researchers conducted literature screening, data extraction and risk of bias assessment independently. Network meta-analysis of the data was performed by Stata 14.0. The Grades of Recommendations Assessment, Development and Evaluation system will be used to evaluate the rank of evidence.

**Results:**

The findings will be reported according to the preferred reporting items for systematic reviews and meta-analyses- network meta-analysis statement. This systematic review and network meta-analysis will summarize the direct and indirect evidence for different kinds of traditional Chinese exercises therapies and to rank these interventions. The results will be submitted to a peer-reviewed journal once completed.

**Conclusion:**

The network meta-analysis was designed to update and expand on previous research results of clinical trials to better evaluate the effectiveness and safety of different interventions of traditional Chinese exercises for type 2 diabetes patients.

**OSF Registration DOI:**

10.17605/OSF.IO/MNJD6.

## 1 Introduction

Diabetes mellitus (DM) is one of the most serious and critical health problems facing the world in the 21st century. DM is a metabolic disorder caused by insufficient secretion of insulin or defective utilization of insulin caused by a variety of etiologies. In diabetic patients, the lack of insulin or defective utilization of insulin keeps glucose in the body at high levels, this long-term high blood sugar would damage many tissues of the body, and increase the risk of cardiovascular disease, kidney disease, and diabetic neuropathy in diabetic patients.^[1]^ Traditional Chinese exercises therapy is the most common exercise method to assist in the treatment of type 2 diabetes mellitus, and it plays an important role in controlling blood sugar and blood lipids and preventing complications.^[2,3]^

Studies have shown that aerobic exercise can improve the sensitivity of peripheral tissues to insulin through a variety of mechanisms, and regulate metabolic disorders in diabetic patients.^[4]^ Compared with other treatments, aerobic exercise is an economical and effective method. It not only has short-term effects but also long-term effects. The short-term therapeutic effect is reflected in the intake of blood sugar and glycogen when exercising, and exercise can also stimulate fat oxidation and storage, the long-term effect is reflected in the improvement of insulin action, blood sugar control, fat oxidation and storage. Studies have shown that participating in aerobic exercise has a certain improvement effect on patients’ mental health, physical symptoms, anxiety and insomnia, social function and depression, and exercise is recommended as a treatment option.^[5]^

Tai Chi is a traditional Chinese mind-body exercise that has been widely practiced in China for many centuries. This exercise has also been applied as a training modality for type 2 diabetes.^[6]^ Other excercises like Baduanjin, Yijinjing, Lizijue, and Wuqinxi have similar therapeutic effects. Those Traditional Chinese Exercises may slow the progression of neuropathy in diabetic patients by improving blood flow to peripheral nerves.^[7]^

However, several different traditional Chinese exercises are often used to treat patients with type 2 diabetes. There are many randomized controlled trials and related systematic reviews and meta-analysis to evaluate the efficacy of various traditional Chinese exercise therapies on type 2 diabetes.^[8–10]^ However, most of these studies are designed to compare with conventional Western medical treatment. Few studies compare the effects of different exercise methods. Therefore, neither clinicians nor type 2 diabetes patients can determine the best option for exercise therapy.

Systematic reviews and meta-analyses are fundamental tools for the generation of reliable summaries of health care information for clinicians, decision makers, and patients.^[11]^ Meta-analyses have usually compared only two interventions at a time, but the need to summarize a comprehensive and coherent set of comparisons based on all of the available evidence has led more recently to synthesis methods that address multiple interventions and these methods are commonly referred to as network meta-analysis.^[12,13]^ So, by using network meta-analysis, this systematic review aimed to systematize the different exercises used to deliver rehabilitation during type 2 diabetes and explore which ones are the most effective.

## 2 Materials and methods

### 2.1 Research registration and results report

This systematic review has been registered on the OSF registration platform and finally we got the Registration DOI: 10.17605/OSF.IO/MNJD6. What's more, this study protocol followed the corresponding guidelines: Preferred Reporting Items for Systematic Reviews and Meta-analyses Protocols^[14]^ and Cochrane Handbook for Systematic Reviews and Meta-Analysis. The Population-Intervention-Comparators-Outcomes-Study design framework (PICOS) was used to guide the eligibility criteria for the review. Ethical approval was not required for this study since the study was conducted based on the data retrieved from previously published randomized controlled trial (RCT).

### 2.2 Inclusion criteria

(1) Randomized controlled trials of Traditional Chinese Exercises therapy intervention in type 2 diabetes patients will be concluded in our systematic review and network meta-analysis.
(2) The subjects have diagnosed by authoritative or recognized type 2 diabetes diagnostic criteria.^[15]^
(3) The observation groups received Traditional Chinese Exercises or Qigong therapy (including Taijiquan, Wuqinxi, Ba Duan Jin, Relaxing Gong, Liu Zi Jue) or Qigong therapy combined with conventional treatment, the control group received conventional treatment or no treatment. The control group is composed of participants who do not receive the experimental treatment.
(4) Outcome measures included glycosylated hemoglobin and fasting blood glucose. secondary outcomes will include health-related quality measurements like body mass index, lipids-related indexes, anxiety and depression scores, and adverse events. It is required to report at least one of the main outcome indicators listed in the included studies.

### 2.3 Exclusion criteria

The following study designs or publication types will be excluded: Literature that is obviously inconsistent with the data in the research, and are suspected of modifying the data without authorization; the clinical results are clear, but the detailed data or important materials are missing, and the original authors cannot be contacted to provide comprehensive information and data analysis cannot be performed.

### 2.4 Database and retrieval strategies

The literature search will be conducted in eight databases including SinoMed, VIP, CNKI, Wanfang, PubMed, Embase, Web of Science and the Cochrane Library from inception to Oct 2021. RCTs that meeting eligibility in published systematic reviews will be identified. We will use the following combined text: “Qigong,” “Qigong therapy,” “TaiChi,” “Ba Duan Jin,” “Yi Jin Jing,” “Liu Zi Jue,” “Wu Qin Xi,” “type 2 diabetes,” “randomized controlled trials,” “RCT.” Database retrieval methods will be adjusted in line with database differences.

### 2.5 Data extraction

Two researchers will perform a duplicate check on the retrieved studies, a preliminary screening of titles and abstracts, and further screening by reading the full text. Data extraction adopts a two-person entry and cross-checking method. In case of disagreement, we will resolve this through discussion between our researchers. The data extraction content will include title, author, disease diagnosis criteria, disease stage, number of patients, average age, gender, research type, intervention measures, course of treatment, outcomes, follow-up, statistical results, and adverse events.

### 2.6 Quality assessment

The bias risk assessment will be completed independently by two investigators using Review Manager (Rev Man, version 5.3) software based on the criteria of the Cochrane Risk of Bias Tool (Cochrane Handbook for Systematic Reviews of Interventions, version 5.1.0).^[16]^ When there is a discrepancy, it will also be resolved through negotiation or by a third investigator. The quality assessment of the included RCTs focused on the following essential information: random sequence generation, allocation concealment, blinding of participants and personnel, blinding of outcome assessment, incomplete outcome data, selective reporting and other sources of bias. The judgment of each item is divided into three levels: low risk of bias, high risk of bias and unclear risk of bias. We will also assess the certainty of evidence contributing to network estimates of the primary outcome through Grades of Recommendations Assessment, Development and Evaluation system.^[17]^

### 2.7 Statistical analysis

**Figure 1:**
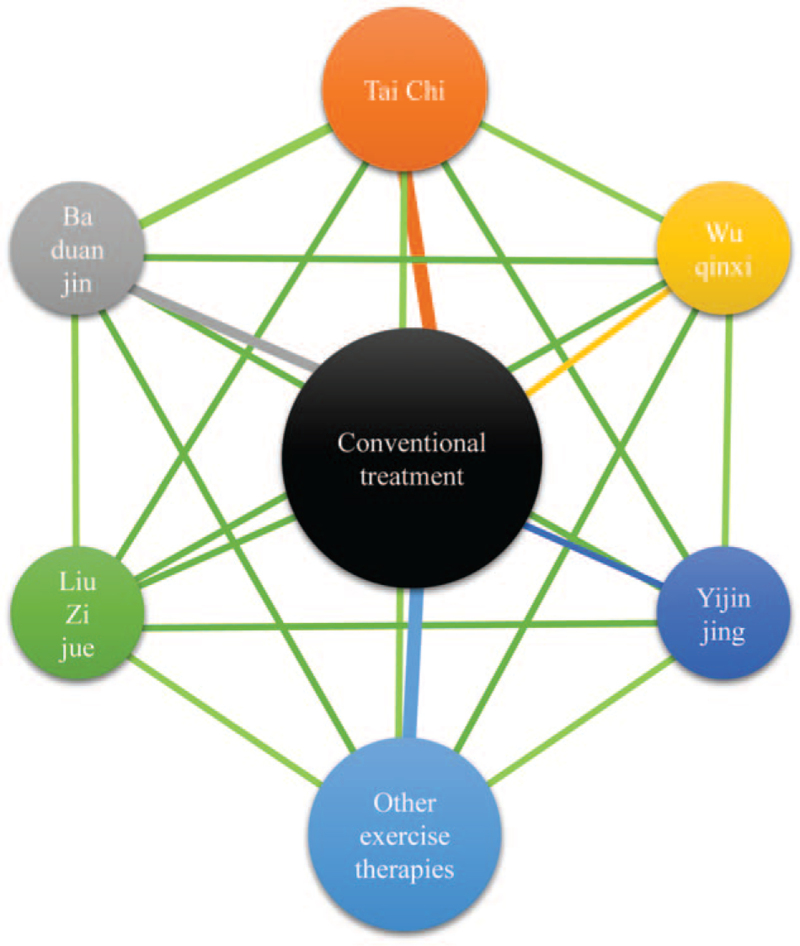
Presentation of network graph on Qigong therapies for type 2 diabetes. The size of treatment nodes reflects the number of patients randomly assigned to each treatment. The thickness of edges represents the number of studies underlying each comparison.

Categorical variable and continuous variable outcomes are respectively used relative risk (RR), mean difference to represent the effect index, and calculate the 95% confidence interval, if there is a three-arm test, it will be split into a two-arm test. Stata/SE 15.0 will be used for the heterogeneity test. If *I*^2^≤50% and *P *≥* *.05, it indicates that there is no significant statistical heterogeneity. Meta-analysis can be used to combine the effect size, if *I*^2^>50%, *P* < .05, it indicates there is significant statistical heterogeneity, and then the Meta-regression, subgroup analysis, and sensitivity analysis will be used to explore the source of heterogeneity.^[18]^ We will use Stata/SE 15.0 to make a network meta-analysis evidence relationship diagram, compare the relationship between the interventions, and use the network plot command to draw the evidence network diagram for the comparison of various treatment measures. If there is a line between the points in the figure, it indicates that the two Interventions have direct comparison evidence, and no connection indicates that there is no direct comparison evidence **(**Fig. 1**)**. Then we will perform the heterogeneity test and inconsistency test. When there is a closed-loop, the consistency of direct comparison and indirect comparison is judged by the inconsistency factor. When the 95% confidence interval of inconsistency factor starting point is 0, the direct evidence and the indirect evidence is consistent.^[19]^ Then the network package of the Stata software will be used to perform a network Meta-analysis, and the results will be sorted, and the surface under the cumulative ranking curve (SUCRA) of each intervention is calculated. SUCRA is a numeric presentation of the overall ranking and presents a single number associated with each treatment. SUCRA values range from 0 to 100%. The larger the value, the better the therapeutic effect. Finally, draw “comparison-correction” funnel plots to determine whether the research has a small sample research effect.^[20]^

## 3 Discussion

There have been evaluations of the efficacy and safety of traditional fitness exercises on the rehabilitation of patients with type 2 diabetes in a stable period. It has been found that traditional fitness exercises can improve exercise endurance, and life quality in patients with stable type 2 diabetes compared with conventional treatment methods. However, a lack of direct comparison results of different exercise therapies, which limits the clinical application of exercise therapy in stable type 2 diabetes patients. On the other hand, perhaps due to the poor quality of the RCT methodology included in this study, no definite conclusion can be drawn, and there is still a lack of strong evidence to recommend the best-ranked Qigong therapy.

Studies have confirmed that long-term exercises like TaiChi, Baduanjin and Wuqinxi improved physical function and psychological state and long-term practice of these exercises helped in the treatment of type 2 diabetes and other chronic diseases. TaiChi is beneficial with respect to physical performance and quality of life in patients with type 2 diabetes.^[7]^ Liuzijue Qigong is helpful for the function of the auxiliary breathing muscles and enhances the flexibility, coordination, and control capacity on neuromuscular limbs to further improve exercise capacity in type 2 diabetes patients.^[8]^ Wuqinxi is one of the most widely practiced forms of Qigong, it has been used to improve physical and psychological health for thousands of years. By mimicking the postures, movements, and bearing of the animals, along with their corresponding breath adjustment, practitioners will experience an activation of the body, so it is suitable for patients with chronic diseases.^[21,22]^

This protocol is designed in accordance with guidelines for network meta-analysis (NMA) protocols. It will be conducted and reported according to the preferred reporting items for systematic reviews and meta-analyses extension statement for NMA. The results of this NMA will be submitted to a peer-reviewed journal once it is completed.

## Author contributions

**Conceptualization:** Huashan Pan.

**Data curation:** Lijuan Zou.

**Funding acquisition:** Huashan Pan.

**Investigation:** Linfeng Lei, Chuifeng Kong.

**Methodology:** Lijuan Zou, Linfeng Lei.

**Project administration:** Huashan Pan.

**Software:** Lijuan Zou, Linfeng Lei.

**Supervision:** Huashan Pan.

**Validation:** Peiying Yu, Jiazhou Li.

**Writing – original draft:** Lijuan Zou, Linfeng Lei.

**Writing – review & editing:** Huashan Pan.
